# LoRaWAN for Smart City IoT Deployments: A Long Term Evaluation

**DOI:** 10.3390/s20030648

**Published:** 2020-01-23

**Authors:** Philip J. Basford, Florentin M. J. Bulot, Mihaela Apetroaie-Cristea, Simon J. Cox, Steven J. Ossont

**Affiliations:** Faculty of Engineering and Physical Sciences, University of Southampton, Southampton SO16 7QF, UK; P.J.Basford@soton.ac.uk (P.J.B.); F.Bulot@soton.ac.uk (F.M.J.B.); mac1g12@soton.ac.uk (M.A.-C.); sjc@soton.ac.uk (S.J.C.)

**Keywords:** Internet of Things, wireless sensor networks, communication networks, smart city, LoRaWAN

## Abstract

LoRaWAN is a Low-Power Wide Area Network (LPWAN) technology designed for Internet of Things (IoT) deployments; this paper presents experiences from deploying a city-scale LoRaWAN network across Southampton, UK. This network was deployed to support an installation of air quality monitors and to explore the capabilities of LoRaWAN. This deployment uses a mixture of commercial off-the-shelf gateways and custom gateways. These gateway locations were chosen based on network access, site permission and accessibility, and are not necessarily the best locations theoretically. Over 135,000 messages have been transmitted by the twenty devices analysed. Over the course of the complete deployment, 72.4% of the messages were successfully received by the data server. Of the messages that were received, 99% were received within 10 s of transmission. We conclude that LoRaWAN is an applicable communication technology for city-scale air quality monitoring and other smart city applications.

## 1. Introduction

Smart city IoT deployments are driving innovations and research in long range low power wireless communication networks. Previous Wireless Sensor Network (WSN) deployments would have used custom hardware and protocols to facilitate communication. The developments in this area have led to a new type of wireless communication network, LPWANs.

These technologies include: LoRaWAN [1], Sigfox [2], and NB-IoT [3], which have reduced the complexity of developing new IoT devices.

The city of Southampton, UK was used as a test bed to evaluate LoRaWAN, one of the LPWAN technologies. This evaluation has required deploying the necessary gateway infrastructure, and assessing its performance. LoRaWAN is used as communication means for the air quality monitors which are currently being deployed in and around Southampton [4,5,6,7]. These air quality monitors log data continuously to local storage and transmit average Particulate Matter (PM) concentrations at regular intervals. These averages allow the air quality in the city to be monitored in near real time. The transmission and receiving times of these messages have been logged and compared. This has enabled the calculation of the percentage of packets successfully received over the duration of the deployment, investigations into the end-to-end delays observed within the network, and any atmospheric effects to be considered.

### 1.1. Low-Power Wide Area Network (LPWAN)

Bardyn et al. [8] state the main characteristics of a LPWAN are: ultra low-power operation, low–cost, no need to wake an end device to maintain network connectivity, ease of deployment of infrastructure nationwide, and secure data transfer. While not included in this list, the long range is also a defining feature of the networks. This means that these LPWAN technologies are not competitors to Bluetooth [9], WiFi [10], Zigbee [11], or other short range wireless communication technologies. A detailed comparison between LoRaWAN, Sigfox and NB-IoT is presented by Mekki et al. [12], and summarised in Table 1. Despite NB-IoT using licensed frequencies compared to LoRaWAN and Sigfox which use the license free Industrial, Scientific and Medical (ISM) band, all technologies have the same problem that the frequencies available in each region differ. This regulatory complexity creates additional challenges when moving devices internationally. All three networks also offer encryption of the payload to prevent eavesdropping of traffic. Security analysis of the LoRaWAN protocol found multiple weaknesses in the LoRaWAN V1.0 specification [13], many of which have been addressed in version 1.1 [14], which is available and soon to be adopted.

LoRaWAN is built on the lower level LoRa protocol, which can be used on its own, but previous work using LoRa for a smart city environment concluded that more robust communication could be achieved by using LoRaWAN on the LoRa physical layer [15]. LoRaWAN is the only network for which it is easy and simple to deploy your own gateway. Both Sigfox and NB-IoT are operated by infrastructure companies, and any additional gateways have to fit within the national operators deployment plan. A personal Sigfox gateway has been announced but distribution is managed by the local network operators who have to be contacted for information [16]. There are multiple vendors offering pre-built LoRaWAN gateways for sale, as well as instructions to make your own custom gateway from a kit of parts.

The ability for users to deploy gateways makes LoRaWAN suitable for city-scale IoT deployments, especially when combined with the localisation and bandwidth capabilities.

#### LoRaWAN

A LoRaWAN deployment can be run totally independently from all other LoRaWAN networks, and this may be beneficial in some commercial or defence use cases; other more open deployments can be build around the existing LoRaWAN community. This community is based around The Things Network (TTN) [17,18], a large LoRaWAN development community and a global deployment community which is rapidly expanding. It is centred on an open and collaborative network providing solutions to facilitate the use of LoRaWAN that enable users to easily use existing gateways to transmit their messages or to add gateways to the network. Data received by TTN is published as an MQTT [19] topic which can be subscribed to by consumers. TTN handles the de-duplication of messages that have been received by multiple gateways simultaneously, further reducing the complexity of implementation. TTN currently has ≈96,000 members, providing ≈10,000 gateways across ≈150 countries.

There is no standard gateway hardware in use on TTN. Any LoRaWAN gateway can be connected to the network. This includes commercially made gateways or those made by users. The different gateway types have different features and make use of different backhaul networks, which is discussed further in Section 2.1.2. Some gateways support a satellite backhaul and work is ongoing to transmit LoRaWAN messages direct to satellites [20]. Although this technology is not currently used in this deployment, it is of interest for future rural deployments.

Localisation of devices based on multilateration of signals is a well established technique [21]. Both Sigfox and LoRaWAN offer support for localisation using different methods. LoRaWAN supports both Timed Difference of Arrival (TDOA) and Received Signal Strength Indication (RSSI) for multilateration of transmissions. This enables devices without Global Navigation Satellite System (GNSS) receivers to provide location aware data streams. The accuracy of the calculated location is dependent on the gateway hardware, the number of gateways that receive the transmission, and the type of the gateway that receives the transmission. All gateways can be used to provide signal strength measurements which can be used for RSSI-based location calculations. For TDOA localisation calculations a (ns) fine grained time stamp is needed for the message. This fine grained time stamp is not available on all gateway nodes because of the specific hardware requirements needed to record the message arrival with the required accuracy. This data can then be fed into the LoRa Cloud location service [22] (previously known as Collos) which uses this data to calculate a position. RSSI gives accuracy of 1000 m to 2000 m compared to TDOA which is in the range 20 m to 200 m [23]. An evaluation of LoRaWAN localisation is presented by Fargas and Petersen [24].

LoRaWAN supports three different modes of operation known as classes: A, B, and C. Each class has different priorities in terms of performance and energy consumption which have been analysed by Cheong et al. [25]. The default for all LoRaWAN devices is to operate in class A, meaning that data can only be received by the end device in a short window after transmission. If the gateway has a Global Positioning System (GPS) receiver then it can be used to provide a beacon broadcast which enables accurate time alignment between end devices, thereby enabling class B which includes scheduled receive windows [14]. Class C requires the end-node to be listening continuously and is designed for either mains–powered devices or transmission of firmware updates to end–nodes during scheduled windows. Operation in any mode requires support from the full hardware and software stack.

The LoRaWAN community is continuously developing, with version 3 of the TTN network stack having over 20 releases in the last year [26]. The rapid development of the network stack means that once an area has gateway coverage additional features can be added through upgrades to the network stack.

### 1.2. LoRa and LoRaWAN Test Beds

Multiple cities have been used to test LoRa and LoRaWAN; some of these deployments use the LoRa Physical (PHY) layer on its own without LoRaWAN on top. Tzortzakis et al. [27] present one such work in which two nodes were deployed at the National Technical University of Athens campus and reported environmental parameters back over a LoRa network. These nodes were 800 m and 500 m away from the gateway. Both the end nodes and the gateway (with General Packet Radio Services (GPRS) backhaul) in this network are solar powered. During the 10 day deployment, 100% of transmitted packets were received.

Lee and Ke [28] have deployed a system that has two major differences to the deployment used in this paper; the network uses the 433 MHz LoRa band, and is a mesh network, unlike the star used in LoRaWAN. By using a mesh rather than a star a node can forward messages received by other nodes onwards to the gateway. This has the advantage of offering greater coverage than can be achieved using a single gateway, but comes at the cost of: increased protocol complexity, increased energy usage on nodes, and less efficient use of available radio bandwidth. During the course of an 8 day deployment consisting of 18 nodes being queried at a 1 min interval, an average of 88.5% packet reception was achieved using a mesh compared to the 58.7% achieved when using a star network. Given the retransmissions required to implement a mesh network, it is not clear how this approach would scale.

Other test beds have used the LoRaWAN protocol on top of the LoRa PHY layer. Pasolini et al. [29] considered both scenarios. During their range tests using LoRa, a maximum range of 2390 m was achieved using Spreading Factor (SF) 12, this poor performance was suggested to be caused by the low height above ground (1.5 m) for the transmitting node. The results from this range experiment were then used as inputs for a simulation to model a planned large scale LoRaWAN deployment to optimise the choice of SF [30]. The results for observed range are significantly below those observed by Basford et al. [7] and Petäjäjärvi et al. [31], but are substantially better than the 1.2 km observed by Loriot et al. [32]. Kulkarni et al. [33] concluded that their tests at a location 0.5 km away from the gateway reached the limit of their deployment; a likely reason for this is the gateway being installed “on a desk in a faculty office”. No details are given as to the elevation of the office. By installing the gateway on a desk it will provide representative data for the indoor experiments performed, but the outdoor measurements should not be compared with data from other deployments with outdoor gateway locations.

Doğan [34] tested LoRaWAN in diverse conditions, in both indoor and outdoor environments, including a tunnel. During the outdoor experiments different power and SF settings were used at four locations across the city with distance from 0.5 km to 3.3 km. For each combination of parameters, 1000 packets were transmitted over the course of 10 days. The performance of the network for each power and SF combination was very location dependent with two locations achieving 100% delivery rate for 78% of the combinations tested. Increasing the SF does not always lead to an increase in packet reception.

Marais [35] deployed a LoRaWAN network for two research projects: a test bed and a water usage monitoring system. The test bed consisted of 18 nodes and the water monitoring project consisted of 34 nodes transmitting every 10 min with Adaptive Data Rate (ADR) enabled. The test bed nodes are located between 0.1 km to 5.2 km away from the gateway. As well as looking at packet delivery rates, the performance of ADR was analysed, with a higher delivery rate being achieved when ADR was disabled [36].

The deployments considered to this point only used a single gateway node. Wixted et al. [37] deployed three gateways across Glasgow. These gateways were used for both coverage mapping and reliability monitoring. The reliability monitoring was performed using acknowledged transmissions over a 1.9 km link. Once initial technical problems were addressed, 98% of messages were successfully received by the gateways.

As well as looking at delivery rates for LoRaWAN networks, there have been studies into the end-to-end delays of LoRaWAN messages. Fernandes Carvalho et al. [38] performed a test using a LoRaWAN transmit node connected to a PC and four separate devices listening to the MQTT application data stream. This experiment was performed as part of the Brescia Smart Living project which covers and area of 80 km${}^{2}$ using over 100 gateways. These gateways then forward the messages to a Patavina NetSuite which manages the LoRaWAN network. Over the course of a day, 1440 messages were transmitted at 1 min intervals with the overall average end-to-end delay being 400 ms to 700 ms, but delays of several seconds were observed.

Pötsch and Hammer [39] performed an analysis of the end-to-end latency of a LoRaWAN network. When the entire LoRaWAN stack was running on a single node, end-to-end latencies of ≈400 ms were observed for SF 7 and 9, increasing to ≈2000 ms for SF 12. When the gateway was separated out and using a Universal Mobile Telecommunications Service (UMTS) connection to the network server, the latency increased to >1000 ms for SF 7 and 9, and nearly 3000 ms for SF 12. The change to a UMTS connection also dramatically increased the standard deviation of the latency of received messages.

The remainder of this paper is structured as follows. Section 2 describes the test bed developed in Southampton; an analysis of the dataset gathered is presented in Section 3; finally, conclusions are presented and areas for future work highlighted in Section 4.

## 2. Southampton City LoRaWAN Deployment

**Figure 1:**
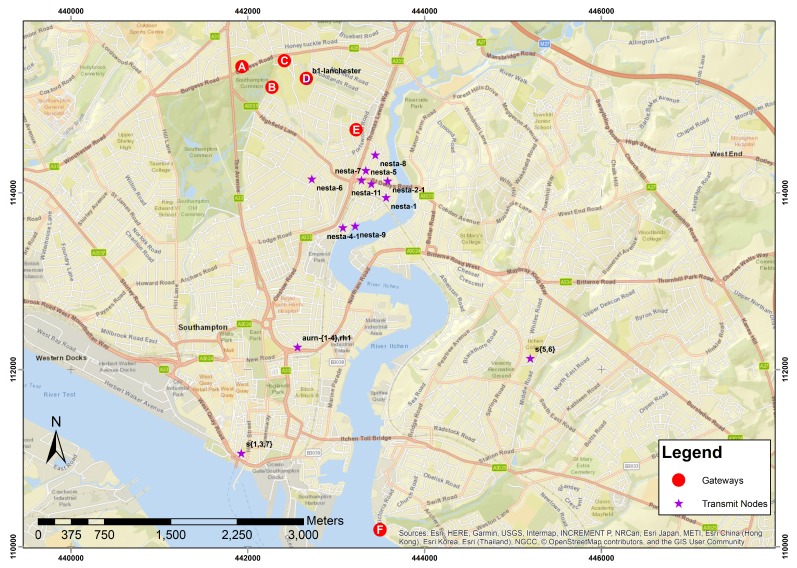
Map showing locations of nodes transmitting LoRaWAN messages and the gateways receiving them. See Table 2 for details of messages transmitted.

The deployment of the Southampton LoRaWAN network used in this paper has been built up over the course of three years. The deployed LoRaWAN network has two primary purposes: (i) providing data connectivity for an on-going air quality monitoring project and (ii) evaluating LoRaWAN for city-scale IoT deployments. Southampton city is situated on the south coast of the UK (see Figure 1), is surrounded by two motorways, and has an airport, commercial dock, and cruise ship terminal. It has a population of ≈250,000 [40]. Air pollution is a major influence on worldwide health, with 6.5 million premature deaths associated with air pollution in 2015 [41]. The air quality in Southampton is an area of research and personal exposure to pollution is of concern [6,42]. This has resulted in the deployment of an air quality sensor network across the city. Not all sensor locations have access to other data networks, making LoRaWAN invaluable for data transfers.

The Southampton LoRaWAN network is intended as a smart city enabler and is made public by forwarding received messages to TTN. The air quality application server then listens to the message stream via MQTT. All data that is received by the application server is backed up to multiple off–site locations daily.

### 2.1. Hardware

All LoRaWAN deployments have two different types of nodes: end-nodes and gateways. These different nodes have different purposes and requirements, meaning they use different hardware and installation environments. LoRaWAN end nodes are designed to primarily transmit messages and the gateways are primarily receivers, constantly listening for transmitted messages. The gateways require an uplink data connection (backhaul) to transmit the messages onwards, usually either a wired/WiFi network connection or a Global System for Mobile communications (GSM) data link, and generally consume more power than end nodes.

#### 2.1.1. End Nodes

The underlying LoRaWAN network used in this publication is summarised in Figure 1 and has been used with multiple different devices to generate multiple datasets. Each type of end–node device is suitable for different applications. The dataset analysed for this publication was generated using Dragino end nodes and an ESM5k sensor [43] as described in Table 2.

The design of the first generation [4] and second second generation [5] of air quality monitor both use the same LoRaWAN hardware: a Dragino LoRa Hardware Attached on Top (HAT) and a Raspberry Pi. This provides the flexibility and processing power needed to interface with the air quality sensors. A major benefit of the Pi Supply LoRa pHAT over the Dragino LoRa HAT is that the operating region can be changed by a software flag. This feature also available on the LoPy, enables the device to roam between 868 MHz and 915 MHz regions without needing hardware or firmware modifications.

The ESM5k temperature sensor is used to record temperature and humidity readings at the same location as the AURN air quality monitors. This provides a comparison between the temperature and humidity inside the air quality monitors and ambient air temperature. The ESM5k was chosen because it uses a temperature and humidity sensor from the same series (SHT3x) as in the air quality monitors. The Siconia nodes could also have been used for this purpose but the specifications of the humidity and temperature sensor are unknown.

#### 2.1.2. Gateways

When deploying a new LoRaWAN gateway, a key decision is whether to build your own or to buy an off–the–shelf gateway. The number of options for gateways has increased dramatically since the start of this deployment in 2016. Commercial grade gateways are rugged, reliable, and have more advanced hardware, but are costly. Another option for gateway construction was using a LoRa interface and a Single Board Computer (SBC) [44]. There are now multiple different LoRa add on boards for SBC; representative examples are shown in Table 3. The lowest cost option to build a custom gateway is to use the same single channel LoRa HAT as used in the end nodes. While single channel operation is perfect for an end device, it is unsuitable for a gateway. Initially, this sort of gateway could be used for a low–cost development environment but has now been superseded by the release of the Things Indoor Gateway; see Table 4. Where a SBC based gateway was used in this deployment it was based around the iMST iC880A because it was the only low–cost LoRaWAN concentrator available. New concentrators have now been released, such as one by Pi Supply. These more recent concentrators have the advantage of supporting direct connection to the SBC without needing an adapter board such as the custom designed Pi-CoT [45]. The main advantages of these home built gateways are: price and flexibility—the ability to design the system connectivity and enclosure to best meet your requirements. The lower price point limits the possibility of including more advanced features, such as ns time stamp accuracy required for TDOA.

The Southampton deployment also makes use of off-the-shelf gateways. These were used for two reasons. The Kerlink iBST base stations shown in Table 4 are used to provide the high–accuracy time stamps needed for calculating locations using TDOA. The deployment also includes a Things Indoor Gateway for evaluation; it has shown be be suitable for home usage. It is an attractive solution for improving coverage inside buildings due to its low cost, but for larger scale deployments The Things Gateway would be better. The Things Outdoor gateway is currently being evaluated and may be used to extend the coverage in Southampton in the future.

The choice of hardware used for a LoRaWAN deployment needs to be made in conjunction with the area in which the network is to be deployed. High or rooftop locations are best for range which means the gateways needs a waterproof enclosure.

### 2.2. Device Locations

The Southampton city LoRaWAN deployment uses different gateway types: Kerlink iBST stations to enable the performing of localisation experiments and multi-channel Raspberry Pi gateways to fill in coverage and enable gathering additional statistics. This has enabled the LoRaWAN network across the city and to perform comparisons of the different gateways.

There are two drivers to deciding on a new gateway location: (i) optimal coverage and (ii) permission for an install. The gateway locations used in this deployment have been obtained by choosing the optimal locations for which permission can easily be obtained.

This approach has led to a dense deployment of gateways around the University campus with four locations (A–D) within a 1 km${}^{2}$; other gateways are located at a residential building (E) and a sailing club (F) shown in Figure 1. Details of the gateways deployed at each location are shown in Table 5. All gateways have external, roof mounted antennas except for the one at location E, where the TTN indoor gateway is located on the ground floor, and the iC880A gateway is located in a roof void.

Locations A and D host both iC880A and Kerlink gateways, and location E has both an indoor gateway and an iC880A gateway. This is due to the progressive deployment of the network: when a new gateway is added to a location, the previous gateway is only removed if it is needed elsewhere to extend coverage; otherwise, it is left in place to provide redundancy. Locations A and D were both initially installed with iC880A gateways and the Kerlinks added at a later date. Location E initially had only the indoor gateway as the infrastructure to install a gateway in the roof space was not present.

The locations of the transmitting devices are shown in Figure 1. The transmission device locations have been chosen based on location requirements from the air quality monitoring projects. This means that their positions are not ideal for monitoring network performance, but do reflect the reality of deploying IoT devices in which the ideal locations may not be available.

All the gateways deployed as part of this work around Southampton forward their data to TTN rather than using a proprietary network, because the advantages of being able to leverage the community services, such as the Semtech LoRa Cloud Geolocation [22] and TTNMapper [46], which are built on top of TTN, currently outweigh the limitations.

## 3. LoRaWAN Message Analysis

The following analysis of LoRaWAN messages was performed on the dataset gathered during the course of the air quality monitoring. That produced a dataset of over 135,000 transmissions. The nodes were deployed at different times due to the availability of sites.

**Figure 2:**
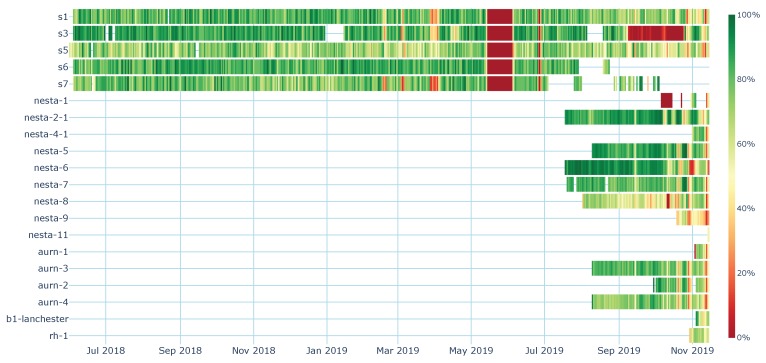
Percentage of LoRaWAN messages received per day for the system as a whole, including: station LoRaWAN transmit, The Things Network (TTN), MQTT receiver, and application storage server). Not all stations were deployed at the same time, and some have been offline for periods of time. The server side data logger failed 14th May–5th June 2019, resulting in a loss of messages. Node rh-1 uses Adaptive Data Rate (ADR) and acknowledgements so is not directly comparable to the other nodes.

The air quality monitors all use the Dragino LoRa HATs for LoRaWAN connectivity (see Table 2 for details of the hardware). These nodes have been configured to use the same SF for all transmissions and do not request receipt acknowledgements. SF 10 was chosen because during initial tests it proven to be the best compromise between time on air and reliability. The node identified as rh1 in Figure 1 and Figure 2 is an off-the-shelf LoRaWAN sensor, ESM5k, used as an additional temperature/humidity sensor within the network. It is set up to transmit every minute with acknowledgements and ADR enabled. In the first 24 h, the sensor adjusted the SF from 7 to 10 and remained on SF 10 validating validates the choice of SF 10 for nodes on which it is hard coded. The differences in configuration between the rh-1 node and the other nodes in the deployment mean that while the data have been included in Figure 2, they have not been included in any of the other analysis of the data.

The air quality monitors s1,3,5,6,7 are configured to transmit every 60 min; the nesta, aurn, and b2-lanchester monitors are configured to transmit every 15 min. All these nodes are configured to log the time at which the message is transmitted. The message is also timestamped on arrival at the server receiving the data. The Real Time Clock (RTC) on all devices are synchronised using Network Time Protocol (NTP) or Pulse Per Second (PPS) when GPS hardware is fitted. The transmit logs are then gathered from the end-nodes and collated on the server. Due to the time required for the message to travel through the TTN servers, the arrival time stamp is not an exact match to the transmit time stamp. The data is processed and if a message from a node is received within 90 s of the transmission of the message it is identified as the same message and marked as successfully received. This approach would not be suitable if the transmission interval is smaller than the matching interval. Any transmission that is not received within the window is marked as failed. For the rh1 node, no logs of transmissions are available, so it is assumed that it transmits as configured, once per minute, giving an expected 1440 messages/day. The message success rate is shown in Figure 2.

### 3.1. Message Delivery Reliability

When considering any form of network communication, the reliability of the link is a key metric. For this study, this is examined by calculating the percentage of messages successfully delivered. The percentage of messages that are received from the transmit nodes each day for each node is shown in Figure 2. Periods shown in white in the image are periods in which no records of transmissions are available. This is typically due to the device awaiting deployment or some other issue preventing transmission of messages (such as power failure). The large vertical red band observed on nodes s1,3,5,6,7 between 15 May 2019 and 4 June 2019 is due to the receiver on the server failing and this not being rectified for an extended period. This outage highlights the importance of having redundancy at all levels of the network and eliminating single points of failure. As the server listens to an MQTT feed a second server listening to the same data feed can be setup to eliminate this single point of failure and reduce the likelihood of this happening again.

Problems in the receiving side of the infrastructure can be identified because they affect all nodes. Other problems are caused by more local issues. The decrease in performance of node s3 between 9 September 2019 and 24 October 2019 was caused by some extremely local environmental conditions, as it did not effect other nodes at the same site. A potential cause for for this would be scaffolding being erected around the node. Node nesta-1 experienced a failure of the LoRa HAT, which meant that while the software executed the call for a transmission, the message was not successfully broadcast.

Across the entire dataset, including the receiver outages, 72.4% of messages transmitted were successfully received across the network. If the outage caused by the server side receiver failure is removed from the dataset the success rate rises to 73.7%. These results fall within the ranges observed by Doğan [34] during their experiments. Marais [35] performed an experiment to calculate the percentage of packets successfully received and observed a success rate of 73.3% over 1000 packets, and 73.5% over 10,000 packets.

### 3.2. Message Delivery Delay

When transmitting messages through an IoT network, it is important to consider how long it takes for the message to be received as it has major implications about a particular communications system suitable for high refresh rate real time data. To generate the dataset used in this publication over 135,000 messages were transmitted and of these 21% were received within 1 s of transmission; see Table 6. The delays observed in this study are greater than those observed by Fernandes Carvalho et al. [38] and Pötsch and Hammer [39]. There are many possible reasons for this. In the work by Fernandes Carvalho et al. [38] the LoRaWAN network server was only responsible for messages from a single city. The LoRaWAN back–end infrastructure used in this study is responsible for all gateways connected to TTN in the Europe region, meaning it has a much higher load. Fernandes Carvalho et al. [38] concluded that the sporadic long delays they observed were caused by the LoRaWAN backend, which supports this theory. Martinez et al. [47] showed that LoRaWAN networks are vulnerable to jamming attacks (such as sending unauthenticated or corrupt packets) with throughput dropping considerably and a processing time that can be up to 100 times higher. While the data available for this study could not allow us to determine exact causes, these two elements could explain the delay and the delivery rate observed in this study.

The figures observed in Table 6 show that when accurate timestamping of data is needed, the time stamp needs to be recorded nearer the edge. If the time stamp is recorded on the end-node itself then the clock has to be kept in sync and the time stamp has to be transmitted over the air. This will increase the amount of data that needs to be transmitted over the network, possibly requiring a lowering of the sample rate to stay within the duty-cycle requirements and fair-usage policies. Another approach is to use the time stamp generated by the first gateway to receive the message as the time of the reading. This does not require the end devices to maintain time synchronisation or the overhead of transmitting the time stamp. The security implications of such a scheme are analysed by Gu et al. [48].

In the air quality use case used to generate the dataset, the data transmitted over LoRaWAN are the 15 min averages (raw data is stored locally), which means a variation of a few seconds in the timestamping can be tolerated, and 99.9% of data is received within 14 s of transmission, making it easy to determine the 15 min window for which the data is valid. The air quality sensors used sample at 1 Hz and any analysis requiring finer grained readings of the PM concentration were performed on the non-aggregated dataset, which was transferred using a traditional Internet Protocol (IP) or GSM network if available. In that use case, the LoRaWAN data were used for health monitoring, data summary, and as a backhaul during potential IP and/or GSM network failures.

### 3.3. Message Scheduling

The LoRaWAN end nodes mainly transmit data hourly or every 15 min, and the TTN received time stamp is used to calculate the payload data time stamp. For example, end nodes transmitting 15 min averages will be timestamped by the TTN received time, rounded to the nearest quarter hour. This is beneficial as there is no need to transmit a time stamp as part of the data payload, saving on bandwidth. Data has to be transmitted and received within the sampling window, which in this case is a minimum of 15 min.

**Figure 3:**
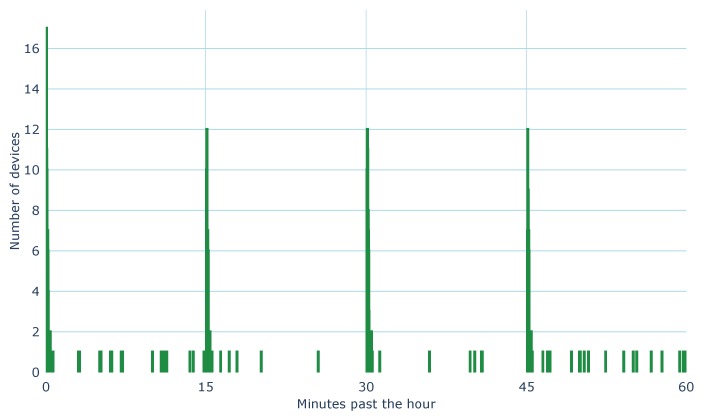
All the end nodes transmit data hourly or more frequently. This figure shows the number of devices that transmit within the same second past the hour (excluding node rh-1). These are not LoRaWAN transmit collisions, but rather, an indication for potential collisions.

Most transmissions have an airtime of under 100 ms, and in an attempt to avoid transmission collisions, there is a short 10 s to 20 s random delay before the data is transmitted. As the number of end nodes in the system increases, the probability of message collisions increases [49,50]. Figure 3 shows the total number of devices that transmitted within the same second past the hour, and it is clear that a better collision avoidance strategy is required. This does not show collisions, but highlights the transmission window and the number of devices that could transmit at a given time; there is a peak every 15 min.

One solution is to change the random delay to just under the sampling period, to ensure delivery within the sampling period, but it is unclear how to manage message retries. Another solution is to include a data time stamp in every message and then queue the messages for transmission. This way messages can be sent whenever retries are permitted, and the data payload always has the correct time stamp, removing the need to fit a time stamp to the data at the server side.

### 3.4. Atmospheric Influence on Message Delivery

Bezerra et al. [51] observed that cold temperatures improved the RSSI and Signal–to–Noise Ratio (SNR) readings during their deployment. These results were observed within a temperature range from −28.7 ${}^{{}^{\circ}}C$ to 24.8 ${}^{{}^{\circ}}C$, giving a range of 53.5 ${}^{{}^{\circ}}C$, with transmissions sent at intervals of between 1 min and 5 min depending on the device. The dataset presented in this paper was collected between −6.1 ${}^{{}^{\circ}}C$ and 29.4 ${}^{{}^{\circ}}C$ for a range of 35.5 ${}^{{}^{\circ}}C$, with five devices transmitting hourly, 12 devices transmitting every 15 min, and one device transmitting every minute. The dataset was analysed to see if any relation between percentage of received packets was in any way influenced by: temperature, rainfall, dew–point, and relative humidity. It was not possible to observe any patterns in the data. This failure to observe patterns could be due to the more limited temperature range or the lower temporal resolution hiding patterns. This does not eliminate the possibility of these parameters influencing the success rate of LoRaWAN transmissions, but shows only that such influences were not observed within this data set.

## 4. Conclusions and Future Work

LoRaWAN has proven to be a useful communication medium for IoT deployments in a city environment. The flexibility of the custom made gateways and the lower price point has enabled more gateways to be deployed than would otherwise have been possible. The use of custom made Raspberry Pi gateways using the iC880A has required the development of a new Printed Circuit Board (PCB) which has been made freely available to the community [45]. This PCB has additional features which provide the required hardware for the operation of class B LoRaWAN devices. While custom made gateways are suitable for testing purposes, the efforts required to build and test these custom gateways mean that when scaling beyond city-scale, the off-the-shelf gateway such as the Kerlink iBTS is the most suitable option.

The long term future of LoRaWAN is not guaranteed; it is the only LPWAN for which is possible to easily deploy your own gateways. The roll of out 5G mobile infrastructure and future standardisation means that other alternatives such as NB-IoT may be able to offer ubiquitous coverage, and will therefore be a strong competitor. Switching from deploying your own network to using national infrastructure changes the cost mode. Deploying your own network has a higher upfront cost, but minimal ongoing costs. Using a national infrastructure has no or very low upfront costs, but a subscription and/or a per-message fee may be charged.

A significant community has been built around the use of LoRaWAN, which will be disrupted by the move to carrier based system. The LoRaWAN community built around TTN offers valuable advice tutorials as well as an annual conference focused on new developments in LoRaWAN and interesting use cases of the technology. LoRaWAN may also prove useful in areas with low or limited internet connectivity [52,53].

This research shows that despite not using optimal gateway locations, a good city-scale LoRaWAN coverage can be achieved. This coverage has been provided by using rooftop locations within the city for which access is available. On average, 72.4% of the messages sent were received, highlighting the need of alternative solutions when data completeness is required. In the air quality use case, IP or GSM networks are used. The PyonAir project [54] overcomes this problem by setting the payload message time to the total number of minutes since the beginning of the month. All messages are added to a queue ready for transmission, messages that are not transmitted within a month expire, and it assumes transmissions are more than one minute apart. This provides a much more robust transmission schedule, and permits message retries. The messages are transmitted randomly throughout the hour, and a tally of the airtime and message count is retained. This way it is easier to utilise bandwidth more efficiently and comply with the LoRaWAN duty cycle and TTN fair usage policy.

This study also revealed that 99% of the messages are received within 10 s of the transmission, which has implications for scenarios requiring high frequency sampling. One solution to improve the coverage would be to evenly spread the message transmissions, but that would come with the added difficulty to time align the readings from the different devices, and would require a coordination of all the users of the network. Further investigation into the causes of the delays and packet loss, such as potential jamming, is required.

This deployment is now generating datasets which can be analysed to evaluate the performance and utilisation of the gateways. Localisation data is being gathered which will enable the accuracy of the multilateration based location calculation to be evaluated. The infrastructure is being expanded with four additional gateways collocated on a multi-story high–rise, to increase the network reliability and extend the area covered. A deployment of 100 air quality LoRaWAN devices [54,55] is scheduled, which will test the scalability of the network and produce a more finely grained dataset both for air quality and for LoRaWAN performance analysis. LoRaWAN is not restricted to just use within the air quality monitoring use case, and other potential uses for the network, including bin usage, parking space occupancy, car counting, and asset tracking, are being explored.

Dataset available from https://doi.org/10.5281/zenodo.3572514.

## Figures and Tables

**Table 1:** Comparison of different Low-Power Wide Area Network (LPWAN) technologies [12]. The standard for each technology is driven by multiple organisations. The modulation schemes used are Chirp Spread Spectrum (CSS), Binary Phase Shift Keying (BPSK) and Quadrature Phase Shift Keying (QPSK); the localisation schemes used are Received Signal Strength Indication (RSSI) and Timed Difference of Arrival (TDOA). Adapted from Johnston et al. [5].

	LoRaWAN	Sigfox	NB-IoT
Frequency bands	Unlicensed	Unlicensed	Licensed
Range (urban)	5 km	10 km	1 km
Range (rural)	20 km	40 km	10 km
Maximum data rate	50 kbit/s	0.1kbit/s	200 kbit/s
Maximum messages per day	Unlimited	140 Up, 4 Down	Unlimited
Modulation	CSS	BPSK	QPSK
Encryption	Yes	No	Yes
Adaptive Data Rate (ADR)	Yes	No	No
Private networks	Yes	No	No
Gateways locations determined by	Anyone	Operator	Operator
Localisation	RSSI & TDOA	RSSI	No

**Table 2:** Comparison of different LoRaWAN nodes. Prices correct as of December 2019; price is for working hardware excluding a power supply, when not included.

	Raspberry Pi & LoRa HAT	Siconia	Pytrack & LoPy	Raspberry Pi & LoRa Node pHAT	ESM5k
Manufacturer	Raspberry Pi & Seeed Studio	Sagemcom	Pycom	Raspberry Pi & Pi Supply	Elsys.se
LoRa hardware	Dragino LoRa	Proprietary	Proprietary	pHAT	Proprietary
Order of power usage	W	mW	mW	W	mW
Built in GPS	Yes	No	Yes	No	No
Customisability	High	None	Medium	High	None
Battery Included	No	Yes	No	No	Yes
Programming Language(s)	Various	JavaScript	MicroPython	Various	None
Enclosure type	None	Waterproof	None	None	Indoor
Multi-region support	No	No	Yes	Yes	No
Cost (USD)	90	40	120	90	100

**Table 3:** Comparison of different Raspberry Pi LoRaWAN gateway solutions. Prices are correct as of December 2019 and exclude suitable external antenna, mounting hardware, and power supplies.

	Dragino Single Channel-Gateway	IMST iC880A	IoT LoRa Gateway HAT
LoRa manufacturer	Dragino	IMST	Pi Supply
Simultaneous channels	1	8	8
Price (USD)	100	250	250
Deployment scale	Desk	Campus	Campus
Uplink	WiFi/Ethernet	WiFi/Ethernet	WiFi/Ethernet
Accurate time stamp	No	No	No
Onboard GPS	Yes	No	No
Waterproof enclosure	No	No	No
Direct connection to Raspberry Pi	Yes	No	Yes

**Table 4:** Comparison of different commercial LoRaWAN gateway solutions. Prices are correct as of December 2019 and exclude external antenna, mounting hardware, and power supplies.

	The Things Indoor Gateway	The Things Gateway	The Things Outdoor Gateway	Kerlink iBST
Simultaneous channels	8	8	≤16	≤16
Price (USD)	80	380	520	2500
Deployment scale	House	Campus	County	County
Uplink	WiFi	WiFi/Ethernet	Ethernet/GPRS	Ethernet/GPRS
Accurate time stamp	No	No	No	Yes
Onboard GPS	No	No	Yes	Yes
Waterproof enclosure	No	No	Yes	Yes

**Table 5:** LoRaWAN base stations located in the city of Southampton, including third party hardware. The Kerlink iBST supports antenna diversity but not all are equipped with dual antenna.

Location	Altitude (m)	Gateway	Antenna	Third Party
A	85	Kerlink iBTS	Procom CXL 900-3LW/I	No
A	85	IMST iC880A	Procom CXL 900-3LW-NB	No
B	45	Kerlink iBTS	Procom CXL 900-3LW/I & Procom CXL 900-3LW-NB	No
C	60	IMST iC880A	RF Solutions FLEXI-SMA90-868	Yes
D	50	Kerlink iBTS	Procom CXL 900-3LW/I & Procom CXL 900-3LW-NB	No
D	45	IMST iC880A	Taoglas OMB	No
E	20	The Things Indoor gateway	Internal	No
E	25	IMST iC880A	CMPLR-ANT415EU	No
F	8	Kerlink iBTS	Procom CXL 900-3LW-NB (Dual)	No

**Table 6:** Delays between transmission and logging of LoRaWAN message. This data only includes messages that were successfully delivered.

Time after Transmission (s)	Percentage of Successful Messages Received
1	21
6	95
10	99
14	99.9
